# The Ubiquitin Code of NODs Signaling Pathways in Health and Disease

**DOI:** 10.3389/fimmu.2019.02648

**Published:** 2019-11-19

**Authors:** Rubén Julio Martínez-Torres, Mathias Chamaillard

**Affiliations:** University of Lille, CNRS, Inserm, CHU Lille, Institut Pasteur de Lille, U1019 - UMR 8204 - CIIL - Centre d'Infection et d'Immunité de Lille, Lille, France

**Keywords:** NOD (nucleotide binding and oligomerization domain) and leucine rich repeat containing receptor (NLR), ubiquitin (Ub), post-translation modification, ubiquitination and degradation, phosphorylation

## Abstract

NOD1 and NOD2 belong to the family of intracellular Nod-like receptors (NLRs) that are involved in the maintenance of tissue homeostasis and host defense against bacteria and some viruses. When sensing such microbes, those NLRs act as hitherto scaffolding proteins for activating multiple downstream inflammatory signaling pathways to promote the production of cytokines and chemokines that are ultimately important for pathogen clearance. In recent years, substantial advances have been made on our understanding of a contextual series of intracellular processes that regulate such group of innate immune molecules, including phosphorylation and ubiquitination. Specifically, we will herein discuss those recently described posttranslational modifications of either NOD1 or NOD2 that fundamentally contribute to the robustness of protective responses within specific tissues through either internal domain association or external interactions with various proteins. From a public health perspective, it is then anticipated that a better understanding how genetic mutations and deregulation of these activating and repressing mechanisms might break down in diseases would open up new therapeutic avenues for humanity.

## Short Summary

### What Are the New Findings?

- LRRK2 and XIAP are involved in the regulation of NOD2 signaling by contributing to form a helical assembly with RIPK2 filaments.
- NLRP12 links bacterial sensing through NOD2 and antiviral immunity using TRIM25.

### How Might It Impact Clinical Practice in the Foreseeable Future?

- The quality control of the NODosome relies on the E3 ubiquitin ligase Parkin, which offers considerable hope for the development of small-molecule activators for the treatment of several diseases, including Crohn's disease.
- The identification of disease-causing TRIM22 variants that affect NOD2 signaling could lead to the development of a new diagnostic tool and could offer novel treatment opportunities for patients with very early onset inflammatory bowel diseases.

## Introduction

The innate immune system contributes to the first defensive actions against trauma and a plethora of microbes. It relies on a wide range of germline-encoded pattern recognition receptors (PRRs) that detect signals from either pathogens or injured host cells (1, 2). The intracellular NOD-like receptors (NLRs) are a relatively new family of PRRs that is related to disease resistance R genes in plants (3). The robustness and outcome of subsequent responses to either injury or pathogenic microorganisms have recently been shown to be influenced by the cooperation of either conventional or unconventional posttranslational modifications (PTMs) of some NLRs, including NOD1 and NOD2 (also referred to as NLRC1 and NLRC2, respectively). Disease-predisposing mutations have been reported in genes encoding for either NOD1 or NOD2 (4). Although it has been well-demonstrated that transcriptional regulation of such sensors is paramount for their function, it is only now that we have started to recognize the relevance of some members of the ubiquitin and TRIM families on positive and negative control of NOD1/2 signaling in health and disease. In this review, we will discuss the most recent reports on how such PTMs influence their processing or deactivation through conformational and compartmentalization changes of either NOD1 or NOD2 within the cell. Specifically, we will describe a number of internal domain association and interactions with endogenous molecules that subsequently control the magnitude and duration of the signal output. For more details on the impact on host fitness of such modifications on other PRRs, we direct the reader to the following outstanding reviews (5, 6).

## The Induced Proximity Model of NOD1/2 Activation

**Figure 1:**
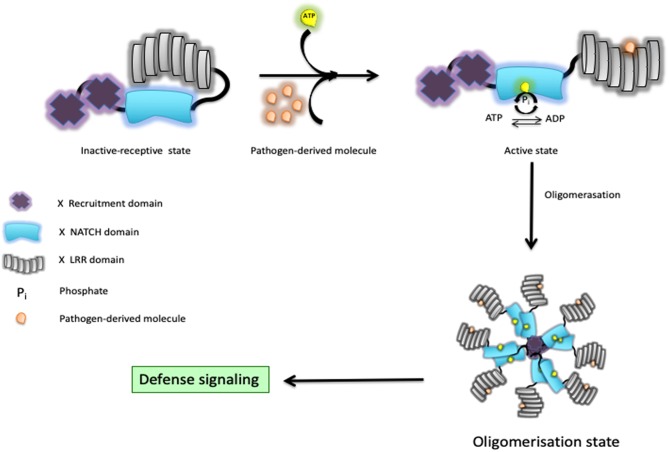
Schematic representation of a Nod-like receptor (NLR) proposed activation model. In response to bacterial muramyl dipeptide (MDP), the NOD2/RIPK2 complex initiates a cascade of events that culminates with the production of chemokines and cytokines through activation of both the MAPK and NF-κB pathways.

The involvement of NOD1/2 in the initiation of innate and adaptive responses has become a topic of great attention in the field of immunology (4). This first led to the discovery that bacteria release muropeptides in their milieu that are sensed by NOD1 and NOD2. NOD1 shows a fairly ubiquitous pattern of expression, whereas NOD2 is more highly expressed in myeloid cells (7–9). As minimal fragments, NOD1 is known to sense bacteria-derived γ-d-glutamyl-meso-diaminopimelic acid (10, 11), whereas the shorter fragment muramyl dipeptide (MDP) is intracellularly detected by NOD2 (12, 13). Consequently, NOD2 responds to the presence of all bacteria, while NOD1 primarily allows the detection of Gram-negative bacteria with the exception of those with an amidation on the meso-diaminopimelic acid, such as *Bacillus subtilis*. Once engaged, they initiate the secretion of several chemokines and cytokines that appear to be context dependent. This relies on well-orchestrated downstream signaling networks that may involve the interferon regulatory factors (IRFs), the mitogen-activated protein kinases (MAPKs), and nuclear factor kappa-light-chain-enhancer of activated B cells (NF-κB) for regulation of host defense and tissue homeostasis. Upon bacterial sensing through their LRR domains, it is thought that the NACHT domain “opens up,” exposes the ATP, and then the molecule adopts “open conformations” in preparation for oligomerization and subsequent signaling (14). Some structural evidence suggests that NOD2 keeps a close inactive-receptive state till it senses bacteria (15). In fact, it presumes that the hydrolysis of ATP to ADP through the NACHT domain can shift the state of the protein from inactive to active and vice versa, and that activity upon bacterial sensing is used as a self-regulatory mechanism to finally shift it to its active state (Figure 1). Indeed, some residues in their NACHT domain might disrupt this self-regulatory mechanism and might render a constitutively active NOD1/2 molecule even in the absence of any detectable bacteria. In short, our understanding of the downstream events is as follows: (i) oligomerized NOD1/2 expose their CARDs for recruitment of RIPK2, forming a NODosome (14, 16); (ii) cumulative binding of phosphorylated RIPK2 (17) to the hetero-CARD complex promotes filament elongation to form a helical assembly (18); (iii) polymerization of its CARD in the presence of ATP stabilizes the active antiparallel dimeric form of RIPK2 (17); (iv) several E3 ligases (such as XIAP, cIAP1/cIAP2, ITCH, PELLINO3, and LUBAC) bind the active form of RIPK2 (19). In the next section, we will summarize recent studies related to the regulation of both signal-induced and homeostatic activation of NOD1/2 at protein level in cells where ubiquitin editing plays a decisive role.

## Nodosome Regulation by the Ubiquitin Code

**Figure 2:**
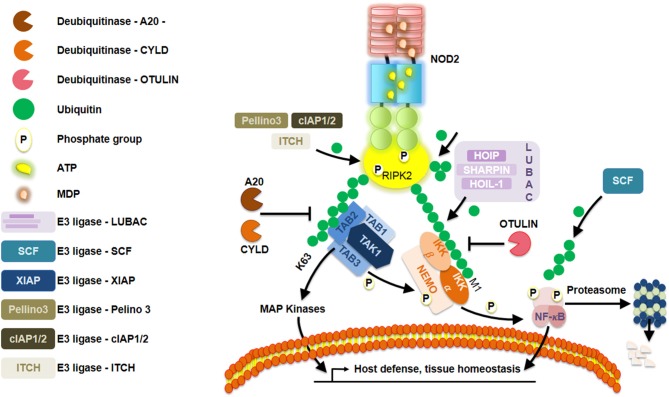
Schematic representation of a Nod-like receptor (NLR) proposed deactivation model. Repression of NOD2 is elegantly orchestrated by a series of posttranslational modifications, such as, ubiquitination and phosphorylation.

RIPK2 becomes lysine63-linked ubiquitinated by XIAP, enabling it to recruit downstream effector proteins (20–22) (Figure 2). Ellwanger et al. (23) recently reported the lack of XIAP, that is, lack of ubiquitination might lead into RIPK2 aggregation. Furthermore, lysine63-linked ubiquitin chains not only are dependent on XIAP but also on other ubiquitin ligases such as cIAP1/cIAP2 (24), ITCH (25), and Pellino3 (26) with RIPK2 as a client protein. Additionally, transphosphorylation also plays a pivotal role in enhancing the robustness of RIPK2-mediated signaling, as recently exemplified by studies on LRRK2 (27). Of equal importance, the addition of methionine1-linked ubiquitin chains to ubiquitinated RIPK2 is performed by the ubiquitin ligase complex LUBAC (28). Among others, three crucial deubiquitinase known as CYLD (29), OTULIN1 (30), and A20 (31) negatively regulate NOD2 signaling by editing ubiquitin chains. Even though many of these events correspond to NOD2, several studies have suggested a similar regulation of NOD1 (32, 33). High-throughput small-interfering RNA screening in HEK293T cells revealed that regulation of actin cytoskeletal dynamics by the COFILIN phosphatase SSH1 influences NOD1 pathway (34). Those findings were further supported by evaluating the response to meso-diaminopimelic acid when knocking down SSH1 expression in different human cell lines and primary human dermal fibroblasts. Using *in situ* proximity ligation assays, they observed that GFP-SSH1 and Flag-NOD1 associated predominantly at F-actin-positive structures. This led the authors to reveal that SSH1 and COFILIN link NOD1 activation to perturbations in the network of actin regulation. Together, they demonstrated that SSH1 acts as a positive regulator of NOD1 signaling. This is reminiscent of what has been first described when studying resistance proteins in *Arabidopsis* that are the counterparts of NLRs in humans (3). In fact, Porter et al. (35) had previously found some genetic evidence that the actin remodeling protein ADF-4 negatively affected RPS4-mediated response in *Arabidopsis*. Studies aiming to understand both the plant R proteins and human NLRs will certainly contribute to a better understanding of these tightly regulated signaling pathways between different cellular contexts. For an overview on the similarities and differences between plant R proteins and human NLR, the reader could refer to an excellent review by Zhang et al. (36).

## Ubiquitin Editing for NOD1 and NOD2 Quality Control

While there are several papers that have discussed the way these NLRs are regulated at gene expression level, different pieces of research have now provided important insights on how their activation/deactivation is achieved by their ubiquitination. Through a series of nuclear magnetic resonance and *in vitro* experiments, Ver Heul et al. (37) measured the chemical shifts perturbations between NOD1 CARD domain and ubiquitin. They found that ubiquitin binds the NOD1 CARD domain through its Tyr88 and Glu84 residues and that lysine63- and linear Met-1-linked ubiquitin but not lysine48-linked were the preferred ubiquitin topology chains. A further elucidation of the crystal structure of ubiquitin bound to NOD1 supported those findings, although a different interaction sites were mapped (38). Similarly, they also found that NOD2 CARDs Ile104 and Leu200 residues were important in binding ubiquitin in the same manner. In that context, they proposed that ubiquitin interfered with the ability of NOD1/2 CARD to associate with RIPK2 as ubiquitin competes for binding to CARD(s) of either NOD1 or NOD2. To further corroborate their hypothesis, they used full-length NOD1/2 wild-type or mutants and found that the NOD1/2-mediated IL-8 response to bacteria was as expected. However, the E84A/Y88R NOD1 and I104R/I200R NOD2 mutants induced a greater ligand-dependent interleukin (IL)-8 secretion while I200L failed to impact on MDP-induced NF-κB activation (33). Besides ubiquitin chain formation on NOD1, there are also few studies on the regulation of NOD2 itself by ubiquitination (i.e., the addition of ubiquitin to a client protein). This has emerged as an important part of receptor activation or processing (39). Specifically, ubiquitination sites have been predicted with high confidence at K436 and K445 by using the composition of k-spaced amino acid pairs. The study described above demonstrated that ubiquitin binds to CARDs of NOD1 and NOD2 and may specifically influence the downstream balance of cell survival processes with that of other metabolic or inflammatory pathways. Together, it appeared that ubiquitin binding may work to attenuate signaling because loss of ubiquitin binding potentiates ligand-dependent NF-κB activation and IL-8 secretion. In other words, ubiquitin seemed to compete with RIPK2 for the NOD1/2 CARDs and disrupt the architecture of the NODosome. This would compromise a negative feedback regulatory loop that is likely engaged when ubiquitin chain formation on the NODosome reaches a critical deactivation point when interacting with ATG16L1 (40, 41). Sorbara Matthew et al. reported that ATG16L1 acts as a NOD1/2-negative regulator independently of its canonical function in autophagosome formation (42). The experiments performed suggested that ATG16L1 diminished NOD1/2-dependent proinflammatory signaling by blocking ubiquitination and subsequent activation of their adaptor protein RIPK2 (42). However, those results are still a topic of debate. This is important as we could argue that ATG16L1 may promote degradation of NOD2 by the proteasome if we take into account the sequence of events that follow bacterial sensing. Understanding how ATG16L1 properly regulates NOD1/2 activation might pave the way to a better understanding of disease pathogenesis (e.g., for Crohn's disease patients bearing variants in *ATG16L1*).

## Implications in Our Understanding of Disease Development

The PARKIN (encoded by the *Park2* gene) has recently been identified to modulate the activity of NOD1/2 signaling. Such RING E3 ligase mediates NOD2 lysine48-linked chain for its degradation in response to endoplasmic reticulum (ER) stress (43). Specifically, it regulates selective degradation of mitochondrial outer membrane proteins through autophagy (44). This work is particularly relevant in Crohn's disease pathogenesis, as previous independent studies have linked either PARKIN (45) or NOD1/2 (46) with ER stress and resistance to epithelial cell death in response to tumor necrosis factor (TNF)-α (47). Using Co-IP studies in Hek293 cells with tagged-PARKIN and -NOD2, they found that they physically interacted, but the exact interacting domains were not specified. Furthermore, they also ectopically expressed both proteins and found that NOD2 is ubiquitinated in a PARKIN dose-dependent manner. The experiments performed using HeLa cells and primary astrocytes showed that PARKIN was upregulated by thapsigargin-induced ER stress in primary astrocytes and that caused a significant reduction of NOD2 levels. They also demonstrated that PARKIN interacted with NOD2 for enhancing its ubiquitination and mediating its degradation. In an earlier study, we learned that the tripartite motif-containing 27 protein (TRIM27) regulated NOD2 through a direct interaction and subsequent ubiquitination. Zurek et al. (48) performed a series of experiments in different cell lines. In that study, they reported that NOD2, but not NOD1, was processed through the proteasomal in an ubiquitin-dependent manner, and TRIM27 was one of the RING-type E3 ubiquitin ligases responsible for NOD2 ubiquitination. Essentially, the PRY-SPRY domain of TRIM27 physically interacted with the NOD2 NACHT domain. Then, NOD2 was tagged with lysine48-linked ubiquitin chains to be degraded by the 26S proteasomal in a TRIM27-dependent manner. Interestingly, they found that the ectopically expressed NOD2 is able to shuttle to the nucleus in a Walker A-dependent manner. That study is interesting because the ubiquitin tagging and subsequent degradation of NOD2 may happen in the nucleus where TRIM27 is primarily located. Once it has accomplished its function, NOD2 is perhaps transported to the nucleus where it can finally be degraded. While more studies are needed to properly dissect this NOD2-TRIM27 interplay, it is clear that TRIM27 is a specific negative regulator of NOD2-mediated signaling under those experimental conditions (49–51). Likewise, TRIM22 has been identified as a positive regulator of NOD2 signaling by contributing to its polyubiquitination. While no effect was observed on either MAVS or RIPK2, a tolerance to MDP was observed in cells bearing a variant of TRIM22 that is associated with the development of very early onset inflammatory bowel disease (51). Of equal importance, there are some other examples in which the interaction with another protein that is not an ubiquitin ligase enables ubiquitination of NOD1/2. For instance, some regulatory molecules such as the suppressor of cytokine signaling 3 (SOCS3) was found to be recruited upon conformational changes of NOD2 that enables ATP hydrolysis (52). That resulted in a reduced affinity to Hsp90, which rapidly dissociated from NOD2. In the absence of Hsp90, SOCS3 associated with NOD2 that became ubiquitinated and underwent proteasomal degradation. The recruitment of a yet unknown ubiquitin ligase by SOCS3 was achieved presumably *via* its SOCS box domain. Consequently, SOCS3 negatively regulates NOD2 signaling. Akin to the previous study, Normand et al. (53) found that NLRP12 directly interacted with NOD2 through its linker region (residues 200–224). Even if the specific NOD2 region involved in the interaction was not reported, they confirmed that NOD2 was found in complex with the heat-shock chaperon Hsp90. Upon NOD2-NLRP12 complex formation, it efficiently sequestered Hsp90 and promotes lysine48-linked ubiquitination and degradation of NOD2 in response to bacterial MDP. Together, they suggested that NLRP12 might interfere with NOD2 signaling in monocyte-derived cells where NLRP12 is primarily expressed and promotes bacterial tolerance. NLRP12 is then a negative regulator of NOD2 signaling and promotes its degradation through a yet-to-be identified ubiquitin ligase. Based on the different studies published today, we could propose that TRIM25, which is negatively regulated by NLRP12 in a protein-protein interaction manner (54), might contribute to the ubiquitination of NOD2. We can speculate that upon viral infection, NLRP12 dissociates from TRIM25, making NLRP12 available for its interaction with NOD2. This might lead to some NOD2 conformational changes, making it available for TRIM25 ubiquitination. Active TRIM25 is then expected to ubiquitinate the RIG-I receptor (54) and possibly NOD2. This could be feasible as NOD2 has been reported to be activated in the response to some single- and double-stranded viruses (55). Noteworthy, some interesting studies have been published, suggesting that ubiquitination and deubiquitylation could play crucial roles on the regulation of NLRC5 (NLR family, CARD domain containing 5). Even though NLRC5 is not a relatively new member of the NLRs, the recent analysis of its regulation has provided some insights into our understanding of its closely related family members NOD1/2 in response to RNA viruses. While being involved in negatively regulating the NF-κB and type I interferon pathway (56–59), NLRC5 has been found to interact with the IKKβ subunit and that NLRC5 lysine 1178 was ubiquitinated with lysine63-linked but not lysine48-linked ubiquitin chains. Furthermore, they identified that TRAF2/6, recruited to the NLRC5-IKKβ complex (58), were the ubiquitin ligases responsible for NLRC5 ubiquitination. Once recruited, TRAF2/6-mediated NLRC5 ubiquitination allowed IKKγ to replace NLRC5 in the complex with IKKα and IKKβ to activate NF-κB. Once NLRC5 lysine63-linked ubiquitin chains were removed by the USP14 deubiquinase, NLRC5 recovered its NF-κB inhibitory function. Thus, this ubiquitin editing on NLRC5 is important to regulate its function in the NF-κB pathway. However, additional investigations are unquestionably required to dissect in greater detail the exact coordinated regulation of that cooperative process with NOD1/2 in health and disease.

## Concluding Remarks

In this review, we have highlighted some of the modifications that specific NLRs must undergo to perform or cease their function. Over the past 20 years, we have greatly advanced our understanding of the activation and regulation of NOD1 and NOD2 that are sophisticated sensors with a contextual versatility in their regulation. Critically, we are now appreciating how ubiquitin plays a crucial role in their regulation that is of utmost importance for understanding how their abnormal control may predispose to development of several human diseases, such as Crohn's disease, allergic asthma, and periodic fever syndromes. While they can self-regulate through a series of internal interaction, it will be extremely useful to obtain the full-length structure to better understand the intra-molecular interaction that governs protein stability and folding and potentially identify druggability regions of those two sensors. Noteworthy, structural (60, 61) and preclinical (62) studies have recently identified some druggable regions within the E3 ligase PARKIN that could be targeted to restore NOD1/2 function to normalcy. Similarly, TRIM27 and TRIM25 seem to be promising targets along with TRIM22, whereby some of its variants were shown to affect NODosome signaling (51). Now, new mass spectrophotometry methods have become more and more powerful for elucidating context-specific protein partners. It is a must to start filling up gaps by elucidating how members of the ubiquitin ligases and kinases may cooperate to allow the development of specific drugs as a promising therapeutic strategy.

## Author Contributions

All authors listed have made a substantial, direct and intellectual contribution to the work, and approved it for publication.

### Conflict of Interest

The authors declare that the research was conducted in the absence of any commercial or financial relationships that could be construed as a potential conflict of interest.
